# Alternative Animal Models of Aging Research

**DOI:** 10.3389/fmolb.2021.660959

**Published:** 2021-05-17

**Authors:** Susanne Holtze, Ekaterina Gorshkova, Stan Braude, Alessandro Cellerino, Philip Dammann, Thomas B. Hildebrandt, Andreas Hoeflich, Steve Hoffmann, Philipp Koch, Eva Terzibasi Tozzini, Maxim Skulachev, Vladimir P. Skulachev, Arne Sahm

**Affiliations:** ^1^Department of Reproduction Management, Leibniz Institute for Zoo and Wildlife Research, Berlin, Germany; ^2^Center for Precision Genome Editing and Genetic Technologies for Biomedicine, Engelhardt Institute of Molecular Biology, Russian Academy of Sciences, Moscow, Russia; ^3^Faculty of Biology, Lomonosov Moscow State University, Moscow, Russia; ^4^Department of Biology, Washington University in St. Louis, St. Louis, MO, United States; ^5^Biology Laboratory, Scuola Normale Superiore, Pisa, Italy; ^6^Leibniz Institute on Aging – Fritz Lipmann Institute, Jena, Germany; ^7^Department of General Zoology, Faculty of Biology, University of Duisburg-Essen, Essen, Germany; ^8^Central Animal Laboratory, University Hospital Essen, Essen, Germany; ^9^Faculty of Veterinary Medicine, Free University of Berlin, Berlin, Germany; ^10^Division Signal Transduction, Institute for Genome Biology, Leibniz Institute for Farm Animal Biology, Dummerstorf, Germany; ^11^Computational Biology Group, Leibniz Institute on Aging – Fritz Lipmann Institute, Jena, Germany; ^12^Core Facility Life Science Computing, Leibniz Institute on Aging – Fritz Lipmann Institute, Jena, Germany; ^13^Department of Biology and Evolution of Marine Organisms, Stazione Zoologica Anton Dohrn, Naples, Italy; ^14^Belozersky Institute of Physico-Chemical Biology, Lomonosov Moscow State University, Moscow, Russia

**Keywords:** Senescence, *Heterocephalus glaber*, *Myotis*, *Nothobranchius furzeri*, *Proteus anguinus*, *Hydra* oligactis, Greenland shark, resistance to cancer

## Abstract

Most research on mechanisms of aging is being conducted in a very limited number of classical model species, i.e., laboratory mouse (*Mus musculus*), rat (*Rattus norvegicus domestica*), the common fruit fly (*Drosophila melanogaster*) and roundworm (*Caenorhabditis elegans*). The obvious advantages of using these models are access to resources such as strains with known genetic properties, high-quality genomic and transcriptomic sequencing data, versatile experimental manipulation capabilities including well-established genome editing tools, as well as extensive experience in husbandry. However, this approach may introduce interpretation biases due to the specific characteristics of the investigated species, which may lead to inappropriate, or even false, generalization. For example, it is still unclear to what extent knowledge of aging mechanisms gained in short-lived model organisms is transferable to long-lived species such as humans. In addition, other specific adaptations favoring a long and healthy life from the immense evolutionary toolbox may be entirely missed. In this review, we summarize the specific characteristics of emerging animal models that have attracted the attention of gerontologists, we provide an overview of the available data and resources related to these models, and we summarize important insights gained from them in recent years. The models presented include short-lived ones such as killifish (*Nothobranchius furzeri*), long-lived ones such as primates (*Callithrix jacchus, Cebus imitator, Macaca mulatta*), bathyergid mole-rats (*Heterocephalus glaber, Fukomys spp.*), bats (*Myotis spp.*), birds, olms (*Proteus anguinus*), turtles, greenland sharks, bivalves *(Arctica islandica*), and potentially non-aging ones such as *Hydra* and *Planaria*.

## Introduction

Most of our current knowledge on mechanisms of aging has been acquired using classical model systems such as mouse (*Mus musculus*) and rat (*Rattus norvegicus domestica*) as well as the common fruit fly (*Drosophila melanogaster*) and roundworm (*Caenorhabditis elegans*). The well-known advantages are easy handling, short generation times, availability of normed strains and standardized husbandry, a wealth of pre-existing information including high-quality and well-annotated genomic and transcriptomic sequencing data (for references see Table 1). The commercial availability of knock-in and knock-out models, cell lines comprising embryonic stem cells, and accessibility to genetic engineering tools such as CRISPR/Cas systems opens the possibility to investigate the underlying biological mechanisms of aging, e.g., by manipulation of related expression patterns (Conn, 2008; Roy et al., 2018).

**TABLE 1:** Overview of alternative animal models.

Common name	Latin name	Taxonomy (Phylum, Class)	Adult body mass	Conservation status ^[1]^	Genome sequenced (size, quality, # protein-coding genes)	Transcriptome available	Life span	Expected life span*	Laboratory husbandry effort
Laboratory mouse	*Mus musculus*	Chordata, mammalia	20–35 g ^[2]^	LC	2.6-Gb; 47x; 30,000 ^[3]^	10 tissues ^[4]^	2-3, max. 3.8 y ^[5]^	0.51 ^[5]^	Small ^[6]^
Laboratory rat	*Rattus norvegicus domestica*	Chordata, mammalia	250-550 g ^[7]^	LC	2.75-Gb; 7x; 22,841 ^[8]^	11 tissues ^[9]^	2-3, max. 4 y ^[10]^	0.32 ^[5]^	Small ^[6]^
Common fruit fly	*Drosophila melanogaster*	Arthropoda, insecta	0.8–1.3 mg ^[11]^	n.n.	120-Mb; whole-genome shotgun; 13,600 ^[12]^	5 tissues; 30 dev. Stages ^[e.g.,^ ^13]^	60 – 80 d ^[14]^	-	Small ^[15]^
Roundworm	*Caenorhabditis elegans*	Nematoda, chromadorea	1μg ^[16]^	n.n.	97-Mb; 6x; 19,099 ^[17]^	4 tissues ^[18]^	14 – 21 d ^[19]^	-	Small ^[15]^
Capuchin monkey	*Cebus imitator*	Chordata, mammalia	2.7-3.7 kg ^[20]^	VU	2.6-Gb; 47x; 20,740 ^[21]^	-	55 y ^[22]^		High ^[23]^
Rhesus monkey	*Macaca mulatta*	Chordata, mammalia	6.3-11.4kg ^[24]^	LC	2.87-Gb; 5x; 21,256 ^[25]^	11 tissues ^[26]^	40 y ^[27]^		High ^[28]^
Common marmoset	*Callithrix jacchus*	Chordata, mammalia	400 g ^[29]^	LC	2.26-Gb; 6x; 21,168 ^[30]^	4 tissues ^[31]^	22 y ^[32]^		Medium to high ^[33]^
Bowhead whale	*Balaena mysticetus*	Chordata, mammalia	50 to > 100 t ^[34]^	LC	2.87-2.91 Gb; 150x; 22,672 ^[35]^	3 tissues ^[36]^	211 y ^[37]^	Longest-lived mammal ^[37]^	Impossible
Mechow’s mole-rat	*Fukomys mechowii*	Chordata, mammalia	345 g (M) 252 g (F) ^[38]^	LC	-	5 tissues ^[10]^	20 y ^[39]^	1.94 ^[39]^	Medium ^[40]^
Naked mole rat	*Heterocephalus glaber*	Chordata, mammalia	33.9 ± 4.9 g ^[41]^	LC	2.7-Gb; > 20 x; 22,561 ^[42]^	10 tissues ^[43]^	32 y ^[44]^	5 ^[45]^	Medium ^[46]^
Brandt’s bat	*Myotis brandtii*	Chordata, mammalia	7 g ^[47]^	LC	2.0 Gb; whole-genome shotgun; 22,256 ^[48]^	3 tissues ^[48]^	41 y ^[49]^	9.8 ^[49]^	Difficult ^[50]^
Budgerigar	*Melopsittacus undulatus*	Chordata, aves	40 g ^[51]^	LC	1.1 Gb; 160x; 15,470 ^[52]^	1 tissue ^[53]^	> 20 ^[54]^	>1 ^[55]^; reproductive life span 5x rats/mice ^[51]^	Small^[56]^
Northern fulmar	*Fulmarus glacialis*	Chordata, aves	650-1000 g ^[57]^	LC	1.14 Gb; 33x; 14306 ^[52]^	-	> 50 ^[51]^; Mean 30 y ^[58]^	Ages more slowly than humans ^[59]^	Medium to high ^[60]^
Japanese quail	*Coturnix japonica*	Chordata, aves	100 g ^[51]^	NT	1.75 Gb; 172x; 30,810 ^[61]^	7 tissues ^[62]^	6 y, max. 11 y ^[51]^	short-lived for birds ^[51]^	Small ^[63]^
Blanding’s Turtle	*Emydoidea blandingii*	Chordata, reptilia	750-1400 g ^[64]^	EN	-	-	75 y ^[65]^	37 ^[65]^	Medium ^[66]^
Painted Turtle	*Chrysemys picta*	Chordata, reptilia	600 g ^[67]^	LC	2.59-Gb;18x; 21,796 ^[68]^	1 tissue ^[69]^	40 y ^[67]^	15-25 ^[67]^	Medium ^[66]^
Axolotl	*Ambystoma mexicanum*	Chordata, amphibia	60–110 g ^[70]^	CR	32-Gb, 7x; 23,251 ^[71]^	16 tissues ^[72]^	10-15 y; max. 25 y ^[70]^	> 1 ^[70]^	Small ^[70]^
Olm	*Proteus anguinus*	Chordata, amphibia	15–20 g ^[73]^	VU	In progress ^[74]^	In progress ^[74]^	Ø 68.5 y, max > 100 y ^[75]^	3 ^[75]^	Difficult ^[73]^
Mudpuppy	*Necturus maculosus*	Chordata, amphibia	50-400 g ^[76]^	LC	-	-	up to 30 y ^[77]^	> 1 ^[77]^	Small ^[78]^
Turquoise killifish	*Nothobranchius furzeri*	Chordata, osteichthyes	3 g ^[79]^	LC	1.24 Gb; 158x; 26,141 ^[80]^	3 tissues ^[79;^ ^81]^	9 (max. 12) weeks ^[82];^ 3-7 months ^[83]^	< 1 ^[66]^ shortest captive lifespan for a vertebrate ^[84]^	Small ^[85]^
Clownfish	*Amphiprion ocellaris*	Chordata, osteichthyes	2-24 g ^[86]^	n.n.	791 - 794 Mb; 3x; 27 420 ^[87]^	Whole-body, 1 tissue ^[87,88]^	> 20 y ^[89]^	>1 ^[89]^	Medium ^[90]^
Greenland shark	*Somniosus microcephalus*	Chordata, chondrichthyes	140 kg ^[91]^	NT	-	-	392 ± 120 y ^[92]^	Longest-lived vertebrate ^[92,93]^	Impossible
Octopus	*Octopus vulgaris*	Mollusca, Cephalopoda	175-3,500 g ^[94]^	LC	2.4-Gb; 76x; 23,509 ^[95]^	5 tissues ^[96]^	1 y ^[97]^		Medium ^[98]^
Red sea urchin	*Strongylocentrotus franciscanus*	Echinodermata, Echinoidea	497.8 ± 32.6 g ^[99]^	n.n.	0.6 Gb; 83x ^[100]^	Developmental ^[101]^	200 y ^[102]^	one of the longest-lived sea urchin^[103]^/animal species ^[104]^	Medium ^[105]^
Green sea urchin	*Lytechinus variegatus*	Echinodermata, Echinoidea	19.5 ± 2.0 g ^[106]^	n.n.	1.3 Gb; 74x ^[100,107]^	Developmental ^[108]^	Average 3 y, max. 4 y ^[109]^	< 1 ^[95]^	Medium ^[105]^
Ocean quahog clam	*Arctica islandica* – Iceland	Mollusca, bivalvia	39-90 g ^[110]^	n.n.	-	2 tissues ^[111]^	Max. 507 y ^[112]^	> 1; Longest-lived non-colonial animal ^[112]^	Small ^[113]^
*Planarian*	*Schmidtea mediterranea*	Platyhelminthes, rhabditophora	17-57 μg ^[114]^	n.n.	782.1 Mb; 60x ^[115]^	All cell types ^[116]^	Non-aging ^[115]^	-	Small ^[117]^
*Hydra*	*Hydra vulgaris/H. magnipapillata*	Cnidaria, hydrozoa	2 × 10^–4^ g ^[118]^	n.n.	0.9-1.05 Gb; 2x; 20,000 ^[119]^	All tissues ^[120]^	Potentially eternal (5% > 1000y) ^[121]^	-	Small ^[122]^

*For mammals: Maximum lifespan divided by expected maximum lifespan (based on the body weight) according to AnAge data and formula (Tacutu et al., 2018); for other species: Different formulas/rationales for expected lifespan as indicated in the respective references. ^[1]^IUCN, 2020; ^[2]^Dutta and Sengupta, 2016; ^[3]^e.g., Waterston and Pachter, 2002; ^[4]^e.g., Harr et al., 2016; ^[5]^Sahm et al., 2018b; ^[6]^National Research Council, 2010; ^[7]^Sengupta, 2013; ^[8]^e.g., Gibbs and Pachter, 2004; ^[9]^e.g., Ji et al., 2020; ^[10]^Sahm et al., 2018a; ^[11]^Kammerer and Young, 1983; ^[12]^Adams et al., 2000; ^[13]^e.g., Graveley et al., 2011; Dobson et al., 2018; ^[14]^Bjedov et al., 2010; ^[15]^Smith et al., 2011; ^[16]^Muschiol et al., 2009; ^[17]^C. elegans Sequencing Consortium, 1998; ^[18]^e.g., Kaletsky et al., 2018; ^[19]^Lapierre and Hansen, 2012; ^[20]^Jack et al., 2014; ^[21]^Orkin et al., 2021; ^[22]^Hakeem et al., 1996; ^[23]^Anderson and Visalberghi, 2010; Schapiro, 2000; ^[24]^Ramsey et al., 2000; ^[25]^Gibbs et al., 2007; ^[26]^Magness et al., 2005; ^[27]^Tigges et al., 1988; Dyke et al., 1986; ^[28]^Schapiro, 2000; ^[29]^Hearn, 1983; ^[30]^Marmoset Genome Sequencing and Analysis Consortium, 2014; ^[31]^Shimizu et al., 2014; ^[32]^Ross et al., 2017; ^[33]^Layne and Power, 2003; ^[34]^Ma and Gladyshev, 2017; Wêsławski et al., 2000; ^[35]^Keane et al., 2015; ^[36]^Seim et al., 2014; ^[37]^George et al., 1999; ^[38]^Scharff et al., 2001; ^[39]^Dammann et al., 2011; ^[40]^Kott et al., 2016; ^[41]^Brett, 1991; ^[42]^Kim et al., 2011; Keane et al., 2014; ^[43]^Yu et al., 2011; Bens et al., 2016; Bens et al., 2018; ^[44]^Ruby et al., 2018; ^[45]^Edrey et al., 2011; ^[46]^Petry, 2003; ^[47]^Munshi-South and Wilkinson, 2010; ^[48]^Seim et al., 2013; Bat 1K, https://bat1k.ucd.ie/, Jebb et al., 2020; ^[49]^Podlutsky et al., 2005; ^[50]^Racey, 1970; ^[51]^Holmes and Ottinger, 2003; ^[52]^Zhang et al., 2014; ^[53]^Künstner et al., 2010; ^[54]^Kamara et al., 2007; ^[55]^Pamplona et al., 2005; ^[56]^Eatwell and Taylor, 2000; ^[57]^Fisher, 1952; ^[58]^Mallory et al., 2012; ^[59]^de Magalhães, 2006; ^[60]^McWilliams, 2008; ^[61]^Kawahara-Miki et al., 2013; Morris et al., 2020; ^[62]^Oster et al., 2020; Finseth and Harrison, 2014; Caetano-Anolles et al., 2015; Marasco et al., 2016; ^[63]^Huss et al., 2008; ^[64]^Congdon et al., 2001; ^[65]^Congdon et al., 2008; ^[66]^Johnson, 2004; ^[67]^Congdon et al., 2003; ^[68]^Shaffer et al., 2013; ^[69]^Manshack et al., 2017; ^[70]^Vladimirova et al., 2003; Farkas and Monaghan, 2015; Vieira et al., 2020; ^[71]^Nowoshilow et al., 2018; ^[72]^Bryant et al., 2017; ^[73]^Holtze et al., 2017; ^[74]^Mulec, 2020; ^[75]^Voituron et al., 2011; ^[76]^Van Devalk and Coleman, 2010; ^[77]^Bonin et al., 1995; ^[78]^Bancroft, 1980; ^[79]^Fumagalli et al., 2020; ^[80]^Reichwald et al., 2015; Valenzano et al., 2015; ^[81]^Petzold et al., 2013; ^[82]^Genade et al., 2005; ^[83]^Platzer and Englert, 2016; ^[84]^Di Cicco et al., 2011; ^[85]^Dodzian et al., 2018; ^[86]^Khoo et al., 2018; ^[87]^Tan et al., 2018; ^[88]^Yang et al., 2019; ^[89]^Sahm et al., 2019; ^[90]^Olivotto et al., 2011; Pryor et al., 2020; ^[91]^Rusyaev and Orlov, 2013; ^[92]^Nielsen et al., 2016; ^[93]^Ste-Marie et al., 2020; ^[94]^Giménez and García, 2002; ^[95]^Zarrella et al., 2019; ^[96]^Zhang et al., 2012; Castellanos-Martínez et al., 2014; ^[97]^Perales-Raya et al., 2014; ^[98]^Iglesias et al., 2004; ^[99]^Warren and Pearce, 2020; ^[100]^Sergiev et al., 2016; ^[101]^Wong et al., 2019; ^[102]^Ebert and Southon, 2003; ^[103]^Francis et al., 2006; ^[104]^Bodnar, 2013; ^[105]^McBride et al., 1997; James, 2007; Taylor et al., 2017; ^[106]^Heflin et al., 2012; ^[107]^Davidson et al., 2020; ^[108]^Hogan et al., 2020; ^[109]^Beddingfield and McClintock, 2000; ^[110]^Stott et al., 2010; ^[111]^Philipp et al., 2012; ^[112]^Butler et al., 2013; ^[113]^Lutz et al., 1981; ^[114]^Oviedo et al., 2003; ^[115]^Grohme et al., 2018; ^[116]^Fincher et al., 2018; ^[117]^Merryman et al., 2018; ^[118]^Fenchel, 1974; ^[119]^Chapman et al., 2010; ^[120]^Wenger and Galliot, 2013b; ^[121]^Schaible et al., 2014; Bellantuono et al., 2015; Klimovich et al., 2018; ^[122]^Lenhoff and Brown, 1970.

**FIGURE 1:**
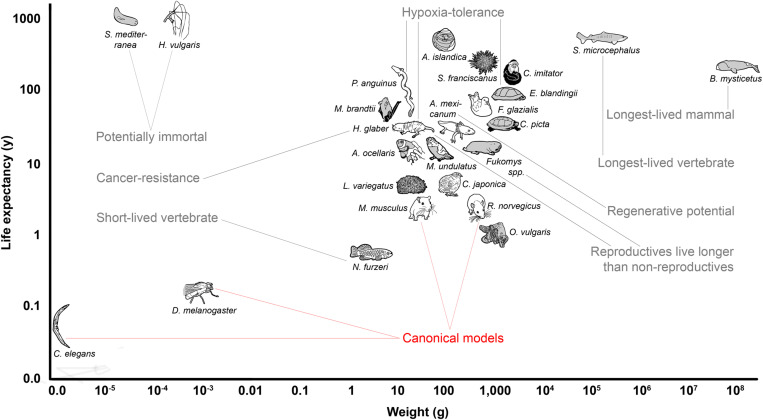
Overview of the body mass to life expectancy relation of canonical and alternative models of aging research. In mammals and many other species, lifespan generally correlates with body weight; therefore, larger species are expected to live longer (compare with Table 1). Note that the canonical models of aging research are all short-lived in relation to body mass. Selected remarkable traits of some of the mentioned species are highlighted.

The drawback of narrowing the view to only a few model species is, however, that it impairs our efforts to gain a broad and general understanding of mechanisms underlying the fundamental biological processes that determine aging. Flies and nematodes with their maximum lifespans of up to a few months (Tacutu et al., 2018) are only to a limited extent representative of the entire animal kingdom in which aging rates vary 40,000-fold (Finch, 1994; Figure 1). Also, mice and rats, with a lifespan of up to 4 years and zebrafishes with up to 5.5 years (Tacutu et al., 2018), are considered short-lived. However, this does not only apply in absolute terms, but also with respect to the known positive correlation between lifespan and body size that explains 63% of the variation in maximum longevity across mammals, birds, amphibians and reptiles (de Magalhães et al., 2007; Fushan et al., 2015; Figure 1). With a life expectancy of only half that expected, mice and rats represent significant negative outliers (Prothero and Jürgens, 1987; de Magalhães et al., 2007). The correlation is highest in mammals and birds, slightly lower in reptiles, and even lower in amphibians, in which longevity further shows a relevant positive correlation with low ambient temperature, nocturnal lifestyle, poison defense, and captivity (Stark and Meiri, 2018). Data regarding fish and invertebrates are scarce, but suggest a similar positive allometric relationship of body weight and lifespan, e.g., among insects (Holm et al., 2016).

It is still unclear to what extent knowledge about molecular aging mechanisms obtained in the canonical model species can be extrapolated to positive outliers such as humans who accordingly live more than 4.5 times as long as expected (Tacutu et al., 2018). For example, life-prolonging effects developed for short-lived species do not necessarily unfold the same beneficial effects in long-lived organisms (Miller and Nadon, 2000). Key components that have been successfully manipulated on a regular basis to extend lifespan in short-lived model organisms are, e.g., the growth hormone/insulin-like growth factor 1 (GH/IGF1) system or the mechanistic target of rapamycin (mTOR) pathway (Laplante and Sabatini, 2012; Junnila et al., 2013). Experimentally prolonged lifespans in short-lived species (Folch et al., 2018) are frequently associated with undesirable side-effects, e.g., decreased fertility or reduced pathogen resistance (Fontana et al., 2010). Finally, the classical model species themselves are equipped with possibly interfering, unique adaptations. For example, mice and rats display specific characteristics such as high fecundity which is associated with a vast range of physiological consequences (Hansen et al., 2014). In addition, research on traditional model organisms often focuses heavily on specific inbred lines, e.g., the mouse strain C57BL/6, which is used in about 90% of biomedical work on this species (Selman and Swindell, 2018). More natural populations show lifespans that in most cases far exceed those conferred by anti-aging interventions on conspecifics of such inbred strains. In addition, there is evidence that wild mouse populations, for instance, already have lower GH/IGF1 signaling - a condition that might thus merely be restored by many classical anti-aging interventions in inbred lines (Miller et al., 2002). The generalizability of findings from short-lived model organisms can and should be validated by studies in positive outliers of the lifespan to body mass correlation or by comparing these outliers with less long-lived species.

Based on those findings from short-lived, canonical model organisms three major categories for hallmarks of aging have been described (López-Otín et al., 2013). ‘Primary’ hallmarks are causally related to molecular damage during aging and comprise genomic instability, telomere attrition, epigenetic alteration, and loss of proteostasis. The ‘antagonistic’ hallmarks, deregulated nutrient sensing, mitochondrial dysfunction, and cellular senescence, have protective and beneficial hormetic effects at low, but detrimental effects at high levels. ‘Integrative hallmarks’ comprise stem cell exhaustion and altered intercellular communication, leading to loss of reserve capacity or resilience.

**FIGURE 2:**
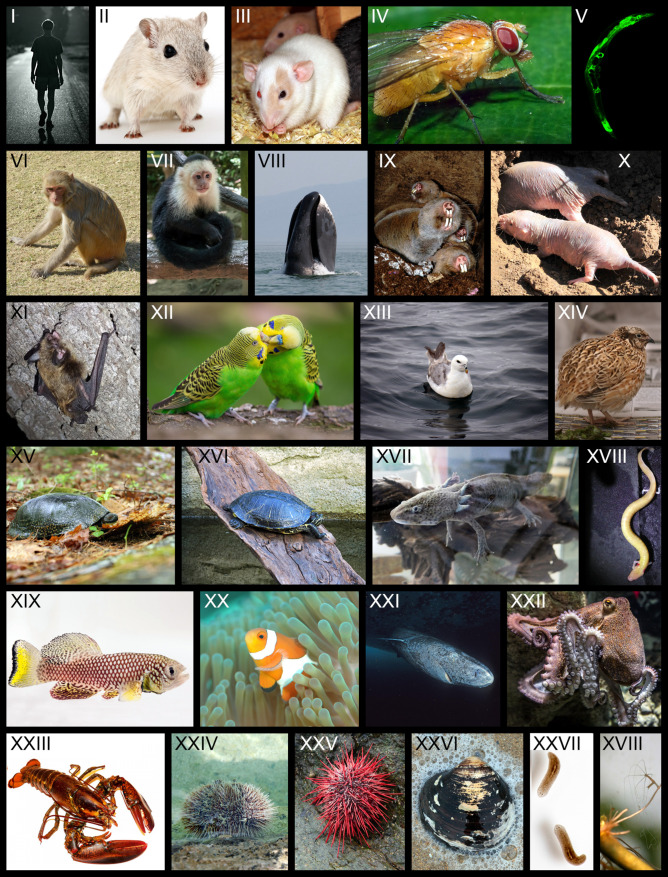
Classical and alternative model species of aging research. In order from **left** to **right**, **top** to **bottom**: I Human (*Homo sapiens*); II Laboratory mouse (*Mus musculus*); III laboratory rat (*Rattus norvegicus domestica*); IV common fruit fly (*Drosophila melanogaster*); V roundworm (*Caenorhabditis elegans*); VI Rhesus monkey (*Macaca mulatta*); VII white-faced capuchin monkey (*Cebus imitator*); VIII bowhead whale (*Balaena mysticetus*); IX Mechow’s mole-rat (*Fukomys mechowii*); X naked mole-rat (*Heterocephalus glaber*); XI Brandt’s bat (*Myotis brandtii*); XII budgerigar (*Melopsittacus undulatus*); XIII northern fulmar (*Fulmarus glacialis*); XIV Japanese quail (*Coturnix japonica*); XV Blanding’s turtle (*Emydoidea blandingii*); XVI painted turtle (*Chrysemys picta*); XVII axolotl (*Ambystoma mexicanum*); XVIII olm (*Proteus anguinus*); XIX turquoise killifish (*Nothobranchius furzeri*); XX clownfish (*Amphiprion ocellaris*); XXI Greenland shark (*Somniosus microcephalus*); XXII octopus (*Octopus vulgaris*); XXIII lobster (*Homarus americanus*); XXIV red sea urchin (*Strongylocentrotus franciscanus*); XXV green sea urchin (*Lytechinus variegatus*); XXVI ocean quahog clam (*Arctica islandica*); XXVII Planarian (*Schmidtea mediterranea*); XVIII hydra (*Hydra vulgaris*). Attributions for used images: V Janine Kirstein; VIII A bowhead whale spyhops off the coast of western Sea of Okhotsk by Olga Shpak licensed under CC BY-SA 3.0; IX Philip Dammann; X, XIII, XVII, XVIII Susanne Holtze; XI Marcus Fritze; XIV; Male Japanese Quail by Ingrid Taylor licensed under CC BY 2.0; IXX Nadine Grimm on behalf of the FLI, Jena; XXI Close up image of a greenland shark taken at the floe edge of the Admiralty Inlet, Nunavut. by Hemming 1952 licensed under CC BY-SA 4.0; XXVI Arctica islandica (Ocean Quahog) by S. Rae licensed under CC BY 2.0; XXVII Anne Schroll on behalf of the FLI, Jena; XVIII Hydra oligactis by Marta Boroń licensed under CC BY 2.0; all other images were taken from Pixabay under the Simplified Pixabay License, links can be provided upon request. This figure was created by Susanne Holtze, IZW Berlin and is licensed under CC BY-SA 4.0.

This review highlights some lesser known, non-canonical and emerging animal models of aging research by giving a short overview on their advantages and limitations, with the aim to facilitate the choice of model species, and to encourage further comparative approaches (Figures 1, 2). By exploring the greater picture, the variety of solutions for exceptional lifespans that originated over the course of evolution can help to identify relevant mechanisms, to gain a more holistic understanding of the processes involved, and eventually may contribute to efforts of extending human healthy aging. This besides underlines the importance of protecting these treasures of biodiversity.

## Mammals

### Primates

Due to their similar aging physiology, humans’ closest living relatives, chimpanzees (*Pan troglodytes*) and bonobos (*P. paniscus*) are of highest interest for research aiming at developing life-extending treatments for humans. However, this advantage is relativized by substantial financial, ethical (Gagneux et al., 2005), legal, and species conservation considerations, and by the impracticability imposed by lifespans of up to 60 years. New World marmosets and Old World macaques are also subject to strict ethical regulations (Mitchell et al., 2021), high cost of housing and long lives, but may embody a suitable compromise. They represent positive outliers from the positive allometric relationship between body weight and lifespan (Figure 1). Total metabolic energy per lifespan, per 1 kg body mass among terrestrial mammals in captivity was found to be highest in monkeys (Atanasov, 2006). The white-faced capuchin monkey (*Cebus capucinus*) has a maximal life expectancy of 55 years (Hakeem et al., 1996), the rhesus monkey (*Macaca mulatta*) of 40 years (Dyke et al., 1986; Tigges et al., 1988), and the common marmoset (*Callithrix jacchus*) of 22 years (Ross et al., 2017). Especially macaques and callitriche share many aging-related diseases with humans, affecting the skeletal, reproductive (Black and Lane, 2002; Colman et al., 2005) and vascular system (Clarkson and Mehaffey, 2009), sensory (Bito et al., 1982; Torre et al., 2004), and cognitive function (Voytko et al., 2009). They also develop diseases such as Alzheimer-like cerebral proteopathy (Heuer et al., 2012; Arnsten et al., 2019), cancer, amyloidosis, diabetes, and chronic renal disease (Tardif et al., 2011), as well as cellular aging (Shimizu et al., 2003). Aging interventions have shown increased longevity due to caloric restriction (Colman et al., 2014), and improved cognitive function due to estrogen therapy in postmenopausal monkeys (Voytko et al., 2009). The effect of rapamycin on non-human primates, a drug showing life-prolonging effects in many model organisms, show basic metabolic effects of long-term treatment on marmosets similar to that seen in short-lived species, with few adverse side effects (Ross et al., 2015; Colman, 2018).

State-of-the-art molecular techniques implemented for monkeys comprise embryonic stem cells of rhesus macaque and common marmoset (Thomson and Marshall, 1997), cloning (Chan et al., 2000) and genetic modification (Wolfgang et al., 2001; Sasaki et al., 2009). The genomes of both species were sequenced (Gibbs et al., 2007; Marmoset Genome Sequencing and Analysis Consortium, 2014) and induced pluripotent stem cells from somatic cells, capable of being differentiated into any cell type established (Liu et al., 2008; Wu et al., 2010). By genetic manipulation and incorporation into embryos, these may serve to generate genetically modified animals.

The disadvantages of using monkeys for aging research may be successfully addressed in the future. The risk associated with monkeys carrying zoonotic diseases, such as hepatitis A, herpes B virus, and tuberculosis (Rothschild, 2015) may be avoided by establishing specific pathogen free populations (Ross et al., 2017). Difficulties arising from the long developmental period and lifespan to perform studies of anti-aging interventions in these models may be solved by the application of modern precise methods of biological age measurements such as the Horvath epigenetic clock (Horvath et al., 2012) which allows for the detection of an intervention effect on a much shorter timescale. And finally, the ethical and conservation-related concerns of research on primates may be altogether circumvented by using the manifold and newly arising possibilities of *in vitro* and stem cell research.

### Fukomys Mole-Rats

African mole-rats (family Bathyergidae) are subterranean rodents that have attracted the attention of gerontologists for more than 20 years due to their generally high longevity. This applies especially to the eusocial *Fukomys* and *Heterocephalus* genera. These genera typically live in extended family groups in which reproduction is monopolized by a few individuals (usually the founder pair). The other family members usually forego their own reproduction within the confines of their natal colonies (Jarvis and Bennett, 1993; Burda et al., 2000). Compared to their body size which lies between that of mice and rats, *Fukomys* species are very long-lived: maximum lifespans of > 20 years have been reported for the Ansell’s mole-rat (*F. anselli;* Dammann and Burda, 2006) and the giant mole-rat *F. mechowii* (Begall et al., 2021), respectively. Possible key factors are enhanced proteasome activity and that *Fukomys* mole-rats exhibit a comparatively high stability of gene expression during aging (Sahm et al., 2018a, 2021). *Fukomys* species are of particular interest to gerontologists because breeders of both sexes live on average approximately twice as long as their non-reproductive conspecifics (Dammann and Burda, 2006; Schmidt et al., 2013). Interestingly, it could be excluded that differences in food intake or behavior cause the different lifespans (Dammann et al., 2011). This feature, probably unique among mammals, offers researchers the opportunity to study highly divergent survival patterns within one genotype, without the inevitable shortcomings of inter-species comparisons. Furthermore, the higher longevity of the breeders is associated with several properties that are the opposite of what is often predicted based on findings from short-lived model species. For example, breeders, contrary to the predictions of the oxidative stress theory of aging, have more advanced glycation end products than age-matched non-breeders (Harman, 2001; Dammann et al., 2012). In addition, anabolic pathways such as insulin signaling and protein synthesis are up-regulated in breeders, which typically shortens the life of short-lived model organisms (Pan and Finkel, 2017; Sahm et al., 2021). Genome-wide comparisons of several long-lived, phylogenetically distinct subterranean rodents including *Spalax galili*, *Heterocephalus glaber*, and *Fukomys damarensis* have associated lifespan extensions with sequence changes in genes related to mitonuclear balance, protein synthesis, autophagy, inflammation as well as resistance to hypoxia, cytotoxins and cancer (Bennett and Faulkes, 2000; Begall et al., 2007; Davies et al., 2015; Sahm et al., 2018b).

Maintaining and breeding *Fukomys* species in captivity is relatively easy, yet time consuming due to generally long generation times and small litters. In fact, gestation and lactation times of ca. 3 month each, and mean litter sizes of 2-3 are typical, while full maturity is usually not reached before the second year of life. Maybe due to these difficulties, only a handful of academic laboratories currently breed *Fukomys* species. As ressources, for comparative studies on aging transcriptome assemblies are available for multiple species including *F. anselli*, *F. mechowii* and *F. damarensis* (Fang X. et al., 2014; Sahm et al., 2018b). A scaffold-level genome assembly is available for *F. damarensis* (Fang R. et al., 2014).

### Naked Mole-Rats (*Heterocephalus glaber*)

Naked mole-rats, despite their small body mass (∼ 32 g; Brett, 1991) are the longest-lived rodents. Their maximal lifespan, exceeding 30 years (Buffenstein, 2005; Grimes et al., 2012; Ruby et al., 2018), is ten times longer than that of mice and five times longer than expected by body mass (Prothero and Jürgens, 1987). Only humans are similarly strong upward outliers among land-living mammals (Austad and Fischer, 1991). Phenotypical aging appears virtually absent for 80% of their lifespan (Edrey et al., 2011) including sarcopenia (Stoll et al., 2016), neurodegeneration (Edrey et al., 2013), disease, and cancer (Buffenstein, 2008; Delaney et al., 2013). Based on sustained fecundity and an apparently age-independent mortality rate, some authors even suggest that the naked mole-rat is the first known example of a non-aging mammal (Buffenstein, 2008; Ruby et al., 2018). Others consider this statement premature due to limited data of older cohorts (Dammann et al., 2019). At the molecular/physiological level, the naked mole-rat shows typical age-related decline, e.g., in activity levels, skin structure (Buffenstein and Jarvis, 2002), liver detoxification pathways (Heinze et al., 2018), lesion and lipofuscin accumulation in various organs (Edrey et al., 2011).

Various, partly overlapping mechanisms were suggested to contribute to longevity and cancer resistance (Shepard and Kissil, 2020), e.g., over-expression of alpha-2 macroglobulin (Thieme et al., 2015), efficient DNA damage repair (MacRae et al., 2015), apoptosis and autophagy of damaged cells (Zhao et al., 2014, 2016; Evdokimov et al., 2018), cytoprotective pathways (Lewis et al., 2015) and proteasome activity (Rodriguez et al., 2014). This list continues with translational fidelity (Azpurua et al., 2013), telomere and epigenome maintenance (Tan et al., 2017; Shekhidem et al., 2019), post-translational deimination (Pamenter et al., 2019), well-maintained splicing regulation (Lee et al., 2020), long non-coding RNAs (Jiang et al., 2016), and a strong innate immune response due to a highly developed myeloid compartment (Hilton et al., 2019; Shebzukhov et al., 2019). The surprisingly low incidence of cancer (Shepard and Kissil, 2020) was associated in particular with early contact inhibition (Seluanov et al., 2009). Accordingly, naked mole-rat cell division would arrest at a comparatively low cellular density mediated by ultra-high molecular mass hyaluronan (Tian et al., 2013, 2015). However, this explanation has been questioned due to non-replicability of central results (Braude et al., 2020; Hadi et al., 2020; del Marmol et al., 2021).

Naked mole-rats show remarkable hypoxia- and hypercapnia-resistance (Larson and Park, 2009) by switching their metabolism to fructose-driven glycolysis (Park et al., 2017) and induction of hypoxia inducible factor 1α and vascular endothelial growth factor A (Xiao et al., 2017). Hypoxia may reduce metabolic rate (Zhang and Lu, 2012) and cause hormetic effects (López-Otín et al., 2013).

Naked mole-rats challenge the free radical theory of aging, predicting reactive oxygen species (ROS) to result in cumulative, irreversible damage causing cellular senescence (Miwa et al., 2008). Despite low body temperature and resting metabolic rate, naked mole-rat mitochondrial oxygen consumption and ROS release are unexpectedly high (Munro et al., 2019). Accordingly, lipid (Andziak and Buffenstein, 2006; Edrey et al., 2014), protein, and DNA oxidation (Andziak et al., 2006) exceed levels of mice, but lack an age-related increase (Andziak and Buffenstein, 2006). Despite low glutathione levels, ROS damage-coping mechanisms such as superoxide dismutase, catalase (Andziak et al., 2005), α-tocopherol activity (Viltard et al., 2019), mitochondrial protein expression (Kim et al., 2011), function, and ROS production are maintained over their lifespan (Holtze et al., 2016; Skulachev et al., 2017; Vyssokikh et al., 2020). Isolated naked mole-rat mitochondria detoxify more ROS than mouse mitochondria via their antioxidative systems (Munro et al., 2019). Also, cell membranes (Hulbert et al., 2006) and arteries are ROS-insensitive (Labinskyy et al., 2006). Genes associated with oxidoreduction, detoxification and mitochondria are under positive selection (Sahm et al., 2018b), and differentially expressed (Yu et al., 2011; Stoll et al., 2016; Heinze et al., 2018).

Colonies of up to 300 individuals of the eusocial naked mole-rats usually contain one breeding pair (Brett, 1991) whose life expectancy exceeds that of non-breeders (Hochberg et al., 2016; Ruby et al., 2018), as in *Fukomys spp.*, offering the unique opportunity to compare intraspecific, e.g., transcriptomic (Bens et al., 2018) differences in aging mechanisms.

The long lifespan, complex social system and lower reproductive rate render research on naked mole-rats more challenging compared to the similarly sized mice. The sacrifice or loss of a naked mole-rat queen may lead to long periods of social instability without reproduction or even the death of many individuals during fighting for successorship.

### Bats (Chiroptera)

Bats can live at least 3 times longer than similarly sized non-flying eutherians (Brunet-Rossinni and Austad, 2004; Wilkinson and Adams, 2019), and Brandt’s bat (*Myotis brandtii*) can live > 41 years, which is up to 9.8 times longer (Podlutsky et al., 2005). Bats reconcile longevity with a high metabolic rate that results in a lifespan-energy expenditure that exceeds that of other mammals by as much as 2 times (Austad and Fischer, 1991). Vespertilionid bats show the greatest longevity-to-body-mass variation among mammals (Munshi-South and Wilkinson, 2010).

Bats’ efficient antiviral immune response (Huang et al., 2019) and tight inflammation control (Kacprzyk et al., 2017) may be primarily adaptations to flight-related rapid body temperature-surges and molecular damage as well as adaptations to crowding (O’Shea et al., 2014). Bats limit self-damaging inflammatory responses by altered interferon regulation (IRF3; Banerjee et al., 2020b), reduced TNF-α- expression (Banerjee et al., 2017), and dampened cellular damage, infection (Ahn et al., 2019) and DNA (Xie et al., 2018) sensing pathways, thereby preventing ‘cytokine storms’ and limiting sterile inflammation, linked to various age-related pathologies (‘inflammaging’; Franceschi et al., 2018). This enhances coexistence with viruses, rendering bats viral reservoir hosts (Banerjee et al., 2020a). Chiropteran cellular homeostatic adaptations may support antiviral mechanisms (O’Shea et al., 2014; Banerjee et al., 2020a). Elevated basal levels of autophagy in bats increase both with age (Huang et al., 2019) and in response to infection (*Pteropus alecto*; Laing et al., 2019). Notwithstanding high protein homeostasis, bat proteasome activity is surprisingly low (Salmon et al., 2009), probably compensated for by higher heat shock protein activity (Chionh et al., 2019) and macroautophagy (Pride et al., 2015). Calcium-dependent neutral proteases (“calpains”) implicated in degenerative processes show low activity in bats, consistent with decreased tissue degradation (Baudry et al., 1986).

Telomere length of long-lived Myotis species is telomerase-independent. Similar to the pattern found in fish (Debes et al., 2016), telomere length correlates with climatic conditions rather than with age or heritability in *Myotis myotis* (Foley et al., 2020; Seeker, 2020). Telomere-associated genes are under positive selection (Morgan et al., 2013) and differentially expressed (Foley et al., 2018). Bats maintain a stable microbiome (Hughes et al., 2018), and fruit eating species display low thyroxin levels (Ifuta et al., 1988). Growth hormone-related peculiarities (Seim et al., 2013) fail to explain longevity in bats (Davies et al., 2014).

In accordance with the oxidative stress theory of aging, bat mitochondrial ROS production is low given their high metabolic rates (*M. lucifugus;* Brunet-Rossinni, 2004). Bat mitochondrial maintenance is enhanced (Jebb et al., 2018; Vyssokikh et al., 2020) and mitochondrial DNA is under positive selection pressure (Nabholz et al., 2008). Bat cells are resistant to ROS-induced apoptosis (Ungvari et al., 2008), hydrogen peroxide (Harper et al., 2007) and to protein oxidation (Salmon et al., 2009). Antioxidant capacity is elevated and DNA-damage is reduced (Conde-Pérezprina et al., 2012) in long-lived (*Desmodus rotundus)* compared to short-lived species (*Myotis velifer*) and in torpid compared to active individuals (*Sturnia lilium;* Wilhelm Filho et al., 2007). Consistent with low cancer incidence (Kitsoulis et al., 2020), DNA repair and signaling pathways are maintained throughout the lifespan (Huang et al., 2019) and two long-lived species (*M. myotis, M. lucifugus*) possess additional copies of the tumor suppressor FBX031 (Seim et al., 2013). The genomes and transcriptomes published by Seim et al. together with further finished and ongoing genome sequencing projects of the approximately 1300 living bat species provide a solid basis for future comparative studies (Jebb et al., 2020).

Lack of established husbandry and captive breeding protocols for many species, as well as the long lifespans remain open challenges. The latter may be addressed e.g., by estimating chronological age of individual bats by using epigenetic signatures (Wilkinson et al., 2021).

### Whales (Cetacea)

Whales are among the longest-lived mammals, with life expectancy comparable to, or exceeding, that of humans, as it is the case of the bowhead whale (*Balaena mysticetus*), which typically populates the cold seas of Greenland and represents the longest-lived extant mammal, being able to survive beyond 200 years of age (George et al., 1999; George and Bockstoce, 2008).

While presenting some age-associated physiological aspects common to other mammals in the senescent phase, such as the age-dependent reduction of fertility observed in humans (Tacutu et al., 2018), whales show an exceptionally low incidence of disease, even at old age. The natural resistance of bowhead whale cells to neoplastic degeneration in particular has been reported (Caulin and Maley, 2011; Magalhães, 2013) and is a classic example of solving Peto’s paradox, the observation that the incidence of cancer does not appear to correlate with the number of cells in an organism (Peto et al., 1975). However, the molecular mechanisms underlying these characteristics of longevity and resistance to age-related diseases (cancer, neurodegeneration, immunosenescence, metabolic dysfunctions) have not been clarified yet.

An interesting phenomenon observed in cetacean genomes is the duplication of genes related to cell cycle control and cancer protection, a phenomenon observed also in the elephant genome that contains multiple copies of the prototypical tumor suppressor TP53 (Sulak et al., 2016) and of further tumor suppressors (Vazquez and Lynch, 2021). Numerous duplicated genes have been described in the bowhead whale genome and some of these are of particular interest in the context of cell damage and survival: for example, the Proliferation Cell Nuclear Antigen plays a fundamental role in repairing DNA damage and is duplicated in the whale genome; both copies are expressed in different tissues and an amino acid substitution is present that alters its ability to interact with other effectors, and therefore its cellular function (Keane et al., 2015). Similarly, the coding sequence of some genes of the mTOR pathway such as LAMTOR1, directly involved in the regulation of various metabolic processes associated with cancer and aging, are modified (Keane et al., 2015). A subsequent study revealed duplication of cancer-related/DNA repair genes, a recurring theme in cetacean evolution. One notable example is UVRAG. This gene plays a dual role in autophagy and DNA repair and is present in multiple copies in the genomes of the sperm whale, North Atlantic right whale, and bowhead whale (Tollis et al., 2019). Consistently, quantitative transcriptome analysis revealed up-regulation of genes for DNA repair and autophagy in the gray whale (Toren et al., 2020). Finally, analysis of coding sequence variation independently identified positive selection on DNA repair/cancer suppression genes (Tollis et al., 2019).

In summary, the insights gained from genomic cetacean studies suggest that molecular adaptations in DNA repair genes played a key role in the evolution of cancer resistance and longevity in these species. Crucial resource requirements for these and presumably future findings were the initial sequencing of the *B. mysticetus* genome (Seim et al., 2014; Keane et al., 2015) which was followed by sequencing of the minke whale (*Balaenoptera acutorostrata*; Yim et al., 2014; Park et al., 2015; Malde et al., 2017), the gray whale (*Eschrichtius robustus*; Moskalev et al., 2017; Toren et al., 2020), the humpback whale (*Megaptera novaeangliae*; Tollis et al., 2019) and the largest mammal, the blue whale (*Balaenoptera musculus*; Árnason et al., 2018). As with other animals that cannot be kept for obvious reasons, a key challenge for the future is to identify experimental approaches that can be used to verify hypotheses derived from such comparative studies with regard to transferability to humans.

## Birds

### Birds (Aves)

Similar to bats, also birds have an increased life expectancy, e.g., more than 50 years in the northern fulmar (*Fulmarus glacialis*), despite higher body temperatures, glucose levels and metabolic rates – as compared to size-matched mammalian species (Holmes and Ottinger, 2003; Harrison and Lightfoot, 2006; Furness and Speakman, 2008; Munshi-South and Wilkinson, 2010). This may be partially explained by their lower reactive oxygen species generation per unit O_2_ consumption and lower oxidative damage levels (Lambert et al., 2007), which has been attributed to reduced activity of mitochondrial complex I (Barja et al., 1994) and their uricotelic metabolism, as elevated levels of blood urates serve as natural antioxidants (Cooper-Mullin and McWilliams, 2016). Among birds, parrots (order Psittaciformes) are especially longlived. A comparative genomic analysis of an endangered parrot species, the blue-fronted Amazon (*Amazona aestiva*) with 30 other bird species has identified longevity-associated genes under positive selection. These are involved in various cellular functions, including telomerase activity, DNA damage repair, cell proliferation control, cancer, immunity as well as anti-oxidative mechanisms (Wirthlin et al., 2018). Cells of another long-lived psittacine, the budgerigar (*Melopsittacus undulatus*) are more resistant to oxidative damage compared to short-lived Japanese quail (*Coturnix japonica*) (Ogburn et al., 2001) and compared to mice (Pamplona et al., 2005). Both budgerigar and Japanese quail are easy to keep and breed, and therefore are highly suitable laboratory species.

Although birds and mammals show similar age-related decline and pathology (Holmes and Ottinger, 2003), their reproductive senescence seems to differ. For example, in the short-lived whinchat, virtually no sign of reproductive senescence could be identified until the age of 4 years, although survival started to decline at an age of only 1 year (Fay et al., 2020). Furthermore, in contrast to mammals, female birds display similar or partially even shorter life expectancies than males (Orell and Belda, 2002; Fay et al., 2020). In a comprehensive study including 339 bird species, it was demonstrated that brain size, independently from body mass, correlates with longevity (Jiménez-Ortega et al., 2020). This supported the “cognitive buffer” hypothesis predicting reduced levels of extrinsic mortality due to improved behavioral flexibility by larger brains (Sol, 2009).

Birds as oviparous vertebrates are widely used for the study of hormone functions on growth and development of the embryo (Groothuis and von Engelhardt, 2005), because the egg represents a closed system almost independent from maternal control once laid. The effects of the hormonal axes on growth, development, and aging can be tested by direct injection, e.g., of thyroid hormones into eggs (Sarraude et al., 2020). The latter increased telomere lengths in collared flycatchers and it was hypothesized that prenatal hormone exposure by this mechanism might set the aging clock in birds (Stier et al., 2020). An age-related decline of telomerase activity was observed in short- but not in long-lived zebra-finches (Haussmann et al., 2004), and in different bird species a negative correlation between telomere shortening and lifespan was found (Sudyka et al., 2016). The annual rate of telomeric repeat shortening appears to be slower in long-lived birds compared to short-lived bird species (Tricola et al., 2018). The delayed senescence of many bird species represents a tremendous potential that can be exploited by comparative, descriptive or conclusive studies.

Despite the many similarities of birds with mammals in terms of cardiovascular anatomy, endothermy, high basal metabolism, and cognitive abilities, these traits have evolved convergently in both phylogenetically distant groups. Therefore, it is plausible to assume avian physiological mechanisms to substantially differ from those of mammals - which nevertheless does not necessarily render them less valuable.

## Fish

### Annual Killifishes (e.g., *Nothobranchius*)

Annual killifishes are adapted to the seasonal alternation of wet and rainy season and inhabit ephemeral ponds that last a few weeks or months (Myers, 1952; Vrtílek et al., 2018a). Duration of the ponds sets a pressure for rapid maturation and an upper limit to the post-hatch lifespan of these fishes. As a result, annual killifishes become sexually mature within a couple of weeks (Vrtílek et al., 2018b) and show rapid age-dependent physiological decline (Cellerino et al., 2016). The contribution of annual killifishes to aging research is two-fold: as a model taxon for comparative approaches based on high-throughput molecular analysis, and as an experimental model to investigate the effects of genetic and non-genetic interventions on lifespan and aging-associated phenotypes (Cellerino et al., 2016; Platzer and Englert, 2016; Poeschla and Valenzano, 2020).

African killifishes of the genus *Nothobranchius* represent a model for parallel evolution of lifespan. The distribution range of the genus overlaps with clines in aridity, and species originating from more humid habitats have a longer lifespan and slower accumulation of age-dependent cellular damage in captivity as compared to species originating from more arid habitats. This pattern is observed in multiple lineages (Tozzini et al., 2013) and was exploited to investigate the genetic architecture of natural lifespan variation (Sahm et al., 2017; Willemsen et al., 2020). Studies of molecular evolution clearly pointed to a prominent positive selection on genes coding for proteins involved in the transcription and translation of mitochondrially encoded members of the respiratory chain as well as proteins involved in the assembly of the respiratory complexes, including nuclearly encoded components of this complex (Sahm et al., 2017). The process that coordinates expression of nuclearly and mitochondrially encoded components of the respiratory chain is known as mitonuclear balance and it has been demonstrated that experimental interference with mitonuclear balance induces life-extension in *C. elegans* (Houtkooper et al., 2013). Notably, the treatment of annual killifish with low dosage of a complex I inhibitor also induces life-extension (Baumgart et al., 2016), demonstrating the complementarity of experimental and comparative approaches in killifishes.

Comparative approaches have also demonstrated that evolution of short lifespan is associated with a relaxation of selection on a large number of genes (Cui et al., 2020).

*N. furzeri* can be cultured in the laboratory with relative ease, in large numbers (Polačik et al., 2016) and with short generation times, as it is the vertebrate with the shortest documented captive lifespan (Di Cicco et al., 2011). Thus, it fills the gap between *C. elegans* and *D. melanogaster*, which are evolutionarily extremely distant from humans, on the one hand, and mice and rats, whose lifespan and housing requirements make life-long investigations unaffordable for many laboratories, on the other. The development of techniques for over-expression and gene knock-outs (Harel et al., 2016) enabled the identification and experimental validation of novel genetic mechanisms of aging. In particular, it was experimentally shown that activity of complex I of the respiratory chain can modulate lifespan (Baumgart et al., 2016), that the microRNA family miR-29 controls neuronal iron homeostasis (Ripa et al., 2017) and cardiac health (Heid et al., 2017) during aging and that decay of proteasome activity is an early event during aging causing loss of stoichiometry in protein complexes (Kelmer Sacramento et al., 2020). Finally, comparative studies of regeneration in killifish and zebrafish revealed evolutionarily conserved enhancers that represent an early response to amputation and are necessary for regeneration to occur (Wang et al., 2020).

In summary, annual killifishes and particularly *N. furzeri*, are the most diffused among the alternative models due to relative ease of housing and breeding. This has contributed to the availability of a number of key resources, such as high-quality genomes and transcriptomes (Reichwald et al., 2015; Valenzano et al., 2015), homo- and heterozygous laboratory strains (Valenzano et al., 2009), genetic manipulation tools (Harel et al., 2016) as well as an increasing knowledge about *N. furzeri*’s ecology and behavior (Thoré et al., 2020). An important challenge for the future is improved standardization, e.g., through the development of husbandry protocols, or time- and cost-efficient test frameworks to determine the effects of chemical compounds on lifespan (Thoré et al., 2020, 2021).

### Long-Lived Fishes

Clownfishes (genus *Amphiprion*) are small (8-15 cm) marine fishes that have adapted to a symbiotic interaction with stinging sea anemones that protect them from predation (Pryor et al., 2020). Clownfishes form bonds with immobile anemones and it is possible to observe the same couple in consecutive seasons (Planes et al., 2009). It is therefore possible to estimate annual mortality which has been shown to be as low as 10% for males (Buston and García, 2007). Estimates of maximum natural lifespan for *A. percula*, based on recapture probability, indicated a lower bound of 22 years (Buston and García, 2007). This estimate is corroborated by observations of living aquarium specimens of *A. ocellaris* and *A. melanopus*, that are over 20 years old (Sahm et al., 2019).

Transcriptome sequencing of clownfishes revealed positive selection in genes related to redox, immunity and mitonuclear balance (Sahm et al., 2019). Further, a comparison with positive selection in naked mole-rats and killifish revealed convergent selection of the mitonuclear balance pathway indicating that selection on the same pathway can modulate lifespan in either direction (Sahm et al., 2019). The genomes of twelve different clownfish species are available and extensive transcriptome information is available for the species *A. ocellaris* (Pryor et al., 2020). Clownfishes are among the very few marine fishes that can be easily cultured in captivity and are currently used as a model to experimentally induce sex reversal (Casas et al., 2016) and pigmentation phenotypes (Salis et al., 2019). Clownfishes have the potential to become the first long-living experimental fish model.

Rockfishes are widely distributed in the Pacific Ocean (Love et al., 2002) and represent an example of adaptive radiation with over 100 species within the genus *Sebastes* (Hyde and Vetter, 2007). The biology and especially life-history of these fishes is known in detail because they are targeted by commercial fisheries and are therefore surveyed by the fishery authorities of United States and Canada. These fishes inhabit a variety of bathymetrics, from intertidal to around 1000 m (Love et al., 2002). A remarkable characteristic of this clade is the large variation in lifespan that ranges from around ten years to more than a century. Interestingly, there is a positive correlation between depth and longevity (Cailliet et al., 2001) and the longest-living species do not appear to undergo reproductive senescence, with fecundity increasing monotonically with age, that would qualify this clade as a model of negligible senescence. Due to the large number of species and large variation in life-history traits, rockfishes hold a great potential for comparative studies and extensive genomic investigations are ongoing (Xu et al., 2019).

The Greenland shark (*Somniosus microcephalus*) inhabits the cold environment of the North Atlantic and Arctic oceans. Paradoxically, this large (up to 5m) shark shows extremely slow annual growth. With a maximum reported lifespan of 392 ± 120 years, determined by radiocarbon measurements, it has been described as the longest-lived vertebrate species known (Nielsen et al., 2016). Remarkably, sexual maturity seems to be reached only after an age of over 100 years.

Although main aspects of Greenland shark biology and life-history are well described (MacNeil et al., 2012), only one molecular study has been conducted to date which tested a possible correlation between exceptional longevity of this species and its specific resistance to oxidative damage (Costantini et al., 2017). However, the assessment of glutathione peroxidase activity and protein carbonyls, as compared to other vertebrate species, did not lend any support for such a relationship.

To exploit the great potential of the Greenland shark for comparative studies on aging, the hopefully early publication of some key resources is required: these include not only the publication of the genome and transcriptome sequences, but also an expanded knowledge of its specific physiology.

## Reptiles

### Turtles (Cheloniidae)

Cheloniidae is a long-lived and successful reptilian family. Perhaps for the same reason, individual turtles protected by a hard carapace and plastron, tend to be long-lived as well. The giant tortoises often live past 100 years and many sea turtle species approach that mark. The genomes of giant Galapagos and Aldabra tortoises have unique genes for DNA repair, inflammatory mediators, telomerase, and nutrient sensing cell-cell inhibition (Quesada et al., 2019), and Leatherback turtles have highly active telomerase (Plot et al., 2012). However, none of these giants would be good candidates for a laboratory model organism because of the challenges of maintaining captive breeding colonies. Fortunately, Blanding’s turtle (*Emydoidea blandingii*) and Painted turtle (*Chrysemys picta*) are more modest sized, therefore more suitable for laboratory study and can live more than 60 years. Even more importantly, long-term field studies exist (since 1953) of Blanding’s Turtle and Painted Turtle (Congdon et al., 2001, 2003) which are especially useful in examining the demographic landscape under which their cellular and molecular adaptations have evolved. For example, long term monitoring of wild Painted Turtle populations has shown that earlier claims that they do not show any signs of senescence (via fertility decline or mortality increase with age) are untrue (Warner et al., 2016). Nonetheless, Cheloniidae do have a number of adaptations to extended hypoxia which may have led to their resistance to the oxidative damage associated with age. Many turtle species spend considerable time in hypoxia, either diving or hibernating in mud. During cycles of diving and resurfacing, mitochondria are forced to alternate between anaerobic and aerobic modes, resulting in production of more reactive oxygen species (Lutz and Prentice, 2002). Turtles deal with this challenge either by upregulating enzymes that clean up these reactive oxygen species, such as glutathione reductase, glutathione synthetase, and glutathione transferase (Willmore and Storey, 1997b), superoxide dismutase (Willmore and Storey, 1997a), necrosis factor kappa B (Krivoruchko and Storey, 2010) or by repairing the damage to lipids and nucleic acids (Willmore and Storey, 1997b). Turtles also have mechanisms for protecting ion channels from reactive oxygen species (Lutz et al., 2003) which may have particular application to age related neurodegenerative diseases in humans. While the genome of Blanding’s turtle has not yet been published, the painted turtle genome is available (Shaffer et al., 2013).

## Amphibians

### Long-Lived Amphibians

As with endothermic species, the lifespan of amphibians as ectothermic organisms correlates clearly with body mass and negatively correlates even more strongly than in endothermic species with body temperature (Keil et al., 2015; Stark et al., 2018). As an additional relevant factor, neoteny (juvenilization, decreased rate of development), which is known to be present in many long-lived species, is widespread among amphibians, especially in the order Caudata. The olm (*Proteus anguinus*) is a neotenic salamander that at a weight of 15-20 g has an average lifespan of 68 years and a predicted maximum lifespan of 102 years. It inhabits water cave habitats in Southern Europe with stable temperatures ranges of 9-11°C (Voituron et al., 2011). At this time, an ongoing olm genome project is not yet completed^1^ and little is known about the mechanisms that contribute to its longevity at the cellular level. It has been reported, however, that in line with its low metabolic rate the olm shows extremely low rates of cell proliferation, e.g., in blood cells (Gredar and Bizjak Mali, 2017). As resources, specific cell populations may be interesting in the context of long-term genome maintenance, for example, the so-called cell clusters of the intestine, which represent slowly proliferating stem cells of intestinal epithelium (Bizjak Mali and Bulog, 2004). Establishing captive *Proteus anguinus* populations is important not only for studies of the mechanisms of aging, but also for preservation of this endangered species (Holtze et al., 2017). Other neotenic amphibians that demonstrate a longer lifespan compared to endotherms of similar body mass include the common mudpuppy (*Necturus maculosus*), a species of the sister evolutionary branch (Zhang and Wake, 2009) that has a predicted lifespan of 34 years, and as a more distant relative, the axolotl (*Ambystoma mexicanum*) that lives for about 20 years (Tacutu et al., 2018). The axolotl itself can be also interesting for the studies on slow aging: researchers have been focused on their unique regeneration capacity for a long time, and now it is clear that the cellular mechanisms controlling epimorphic regeneration are also involved in the development of cancer and aging (McCusker and Gardiner, 2011; Vieira et al., 2020). For example, the expression of well-known oncogenes (*Foxo1, Myc, Kazald1*, etc.) is markedly increased in axolotl blastemas (tissue of regenerating limb) at the early stages of wound healing (Stewart et al., 2013; Bryant et al., 2017). At the same time, low incidence of cancer is characteristic for this species (Vieira et al., 2020). Recent work on single-cell sequencing of blastemas highlights the importance of different cell types involved in limb regeneration. For instance, highly represented regulatory myeloid and T cells possibly contribute pro-regenerative over oncogenic molecular context in growing blastemas (Leigh et al., 2018). Aside from abundant transcriptomic data, gene-editing methods are largely described in the axolotl model (Khattak and Tanaka, 2015; Kuo and Whited, 2015; Fei et al., 2017, 2018). Altogether it makes *A. mexicanum* a powerful system for searching for genetic or cellular factors which define differences between similarly built regeneration and tumorigenesis molecular programs (Boilly et al., 2017), possibly providing unexpected insights into aging research.

The assembly of salamander genomes is challenging because of its large size and excessive number of repetitive regions (axolotl genome; Nowoshilow et al., 2018). On the other hand, transcriptomic data is available for a number of salamanders (Joven et al., 2019), including axolotl (Stewart et al., 2013; Bryant et al., 2017) and the genome sequencing of further species such as the olm and Chinese giant salamander (*Andrias davidianus*) is currently on the way.

## Invertebrates

### Cephalopods

Coleoid cephalopods (octopuses, squids) have attracted the interest of gerontologists because of their highly variable lifespans (Schwarz et al., 2018). However, the significant variation in cephalopod lifespans remains little studied (Schwarz et al., 2018). For instance, littoral (*O. minor, O. bimaculoides*) and shallow water octopuses (*O. vulgaris*, *O. maya*) usually live for 1-2 years (Rodríguez-Domínguez et al., 2013; Perales-Raya et al., 2014; Kim et al., 2018), while the estimated lifespan of cold-water (*Pareledone charcoti*) or deep-sea octopuses (e.g., *Graneledone boreopacifica*) is 7-10 years (Schwarz et al., 2018). Both short-lived and long-lived octopods perform one cycle of reproduction, and die after hatching of the eggs, a phenomenon called phenoptosis – programmed death of the entire organism (Skulachev, 2012). Maximum lifespan in these species thus appears to be strictly tied to the duration of puberty and subsequent mating. Interestingly, parental behavior in octopods depends on a specific organ, the optic gland. During brooding the optic gland induces fasting by expression of steroids and insulin signaling regulators (Wang and Ragsdale, 2018). The removal of optic glands prolongs life and prevents parental care in octopuses (Wodinsky, 1977; Wang and Ragsdale, 2018). Furthermore, octopuses are the most highly organized invertebrates: their large neural and sensory systems as well as complex behavior keep gaining researchers’ attention (Huffard, 2013).

A large number of studies show a correlation between environmental temperature and growth rate in octopod embryos and larvae (Hoving and Robison, 2017; Schwarz et al., 2018; Braga et al., 2021). Thus, cold water species apparently have a prolonged maturation period. On the other hand, long-lived octopuses exhibit parental care and guard their egg-clutches throughout all embryonic stages; therefore, the period of brooding lasts longer (Robison et al., 2014). The molecular basis of these adaptations in long-lived octopuses remains largely unknown, but may be related to effective antioxidant systems. For instance, the short-lived *O. vulgaris and O. tehuelchus* demonstrate flexibility of antioxidant systems in different environments (Fassiano et al., 2017; Sillero-Ríos et al., 2018).

Laboratory housing conditions are well described for a variety of octopod species (Van Heukelem, 1977; Iglesias et al., 2004; Semmens et al., 2011), and keep improving. This opens the path to address novel biological questions, e.g., regarding evolutionary developmental biology (Maldonado et al., 2019). Genomic (Albertin et al., 2015; Kim et al., 2018; Zarrella et al., 2019) and transcriptomic (Zhang et al., 2012; Castellanos-Martínez et al., 2014) data so far are available only for short-lived octopuses. The combination of the rare semelparous reproductive strategy and their cognitive abilities render octopuses interesting alternative models of aging. To exploit this potential for comparative studies on aging, some key resources, such as genomes of longer-lived species and an expanded knowledge about their physiology need to be established.

### Hydra

In demographic studies with species of the genus *Hydra* (freshwater polyps) it was shown that several of them have constant low mortality and high fertility rates over long periods of time (Martínez, 1998; Jones et al., 2014; Schaible et al., 2015) leading to the widespread view that these animals are non-senescent (Schaible et al., 2014; Bellantuono et al., 2015; Tomczyk et al., 2015; Klimovich et al., 2018). These studies predicted that at least 5% of the *Hydra* population would reach an age of more than 1000 years under laboratory conditions. It is assumed that this high potential for longevity has largely evolved as a by-product of *Hydra’s* regenerative capacity which allows these animals to fully restore any part of their body within days in the event of injury (Vogg et al., 2019b). When cut into pieces, a complete animal can regenerate from each piece, in extreme cases even from excisions corresponding to 1% of the original animal (Shimizu et al., 1993). The approximately 1 cm long body consists of essentially a digestive tube, with the mouth/anus on one side surrounded by the tentacles arranged in a circle, and a foot on the other. An adult polyp has at maximum about 100,000 cells of twelve cell types and three distinct stem cell lineages (Tomczyk et al., 2015). The regenerative ability is the result of a high proportion of stem cells, which ensures constant self-renewal with a cell cycle of 1-4 days depending on the stem cell lineage (Bosch and David, 1984; Bode, 1996). Driven by the continuous proliferation of stem cells, under optimal conditions *Hydra* usually reproduces asexually through budding. A reduction of the water temperature from 18° to 10°Celsius results in a time-dependent mortality increase among *H. oligactis* (Yoshida et al., 2006) suggesting that this measure is sufficient to induce aging. This offers the apparently unique opportunity to compare molecular signatures of non-aging and aging-like phenotypes within a single species (Bellantuono et al., 2015). Both annotated genomes (*H. vulgaris, H. viridissima* and *H. oligactis*) and a transcriptome (*H. vulgaris*) are available (Chapman et al., 2010; Wenger and Galliot, 2013a; Vogg et al., 2019a). Furthermore, high-resolution gene expression data sets are available, e.g., with respect to regeneration time series (Petersen et al., 2015) or on single cell level (Siebert et al., 2019). Despite being taxonomically more distant, *Hydra* shares as many genes with humans as *D. melanogaster* (both about 6000) and even more than *C. elegans* (about 4500) (Wenger and Galliot, 2013a). Particular attention has been paid, e.g., to the evolutionarily conserved transcription factor FoxO, which is believed to play a possible key role in maintaining the inexhaustible self-renewal capacity of *Hydra* stem cells and thus in apparent biological immortality (Boehm et al., 2012; Bellantuono et al., 2015; Martins et al., 2016). Generations of stable transgenic animals have been used several times both for overexpression and knockdown experiments (Wittlieb et al., 2006; Gee et al., 2010; Vogg et al., 2019a). Also, inducible RNA interference based gene silencing approaches can be applied (Watanabe et al., 2014; Vogg et al., 2019a). *Hydra* is easy to keep, both individually, e.g., in Petri dishes, and en masse, e.g., in glass bowls, at water temperatures of 18°Celsius and feeding them every one to three days. One of the key challenges for the future will be to transfer the knowledge gained from this evolutionarily distant species to humans, e.g., in the field of regenerative medicine.

### Long-Lived Marine Non-colonial Invertebrates

Marine invertebrate species can be found in different invertebrate groups (Mollusca, Crustacea, Echinodermata), with different ecological niches, inhabiting a range of geographical zones at different temperatures and depths (Bodnar, 2009; Murthy and Ram, 2015; Ram and Costa, 2018). Among the features that unite these organisms are delayed growth and relatively low extrinsic mortality due to protective structures (shell, spines).

The most widely studied long-lived bivalve clam is the ocean quahog of the North Atlantic (*Arctica islandica*), the highest documented lifespan of which reaches at least 507 years. It should be noted that this abnormally high lifespan is characteristic for Icelandic population of *A. islandica*, while the Baltic Sea and White sea populations have a maximum lifespan of 30-50 years (Basova et al., 2012; Gruber et al., 2015). Ocean quahog is often mentioned as a candidate for negligible senescence: individuals develop rapidly until sexual maturation, then the growth ceases, and the organism exhibits no signs of senescence for decades. The same molecular dynamic was established for the antioxidant system: young ocean quahogs exhibit high antioxidant capacity compared to other bivalves, but then, at age of 30, the mitochondrial citrate synthase as well as the catalase and glutathione stabilize at lower post-maturation levels and show no tendency for further decline with age (Abele et al., 2008). A more recent study of mitochondrial membrane lipidome demonstrated lower peroxidation indexes in long-lived *A. islandica* than in short-lived bivalve species (Munro and Blier, 2012). However, an intraspecific study of six *A. islandica* populations suggests that lipid membrane composition does not correlate with longevity.

Highly variable mean telomere length and activity of telomerase, which is continuously expressed in different tissues, were investigated in the longest- versus shortest-lived populations of *A. islandica*. But the fact that equal telomere dynamics are shared between *A. islandica* populations with extremely different maximum lifespans indicates that constant telomerase activity apparently contributes to longevity but does not determine it (Gruber et al., 2014). Thus, unique factors defining the ocean quahog’s longevity remain to be discovered. *A. islandica’s* genome has not been published so far but a mitochondrial genome is available (Glöckner et al., 2013).

Genome sequencing of the red sea urchin (*Strongylocentrotus franciscanus)*, which reaches a lifespan about 200 years (Tacutu et al., 2018), and of the green sea urchin (*Lytechinus variegatus*) allowed for a comparison between closely related long and short-living species. Mitochondrial proteins, lipid transport proteins (ApoB), proteins involved in amyloidogenesis (PSEN1) and the system of telomere maintenance (particularly, TERT) of *S. franciscanus* were enriched in amino acid substitutions which are specific for a long-lived species (Sergiev et al., 2016).

*Homarus* is a genus of lobsters whose members are estimated to reach lifespans of up to 50 years in the wild (Wolff, 1978) and up to 100 years in captivity (Bowden et al., 2020). They grow indefinitely, are able to regenerate limbs even at high ages and old animals may be more fertile than young ones (Klapper et al., 1998; Koopman et al., 2015; Tacutu et al., 2018). Unlike in most other animals, telomerase activity is high also in adults and in all tissues (Klapper et al., 1998).

Overall, marine invertebrates represent powerful models for aging research, combining ease of chronological age determination if captured in the wild and convenience of culturing in captivity. Further studies on longevity using these models require development of methods, such as cell culturing and gene manipulation, to be adapted for the marine invertebrate species (Ram and Costa, 2018).

### Planaria (Tricladida)

Like *Hydra*, planarian flatworms are described as theoretically non-aging based on their regeneration ability (Sahu et al., 2017; Johnson et al., 2019). Although they are complex organisms with, for example, a central nervous system, a digestive tract and eyes, a complete individual can grow from small excised body fragments of almost any tissue. A high proportion of partly pluripotent adult stem cells (about 25-30% of all cells), called neoblasts, play a decisive role in this ability (Reddien and Sánchez Alvarado, 2004). The neoblasts incorporate positional signals into the processes of proliferation and differentiation and thus guarantee the almost perfect scaling of tissues and organs during regeneration (Reddien, 2018). Based on this, *Planaria* evolved asexual reproduction by fission as an alternative to sexual reproduction. The worm splits horizontally into a head and a tail part, each then restoring the missing part (Malinowski et al., 2017). Some species even reproduce exclusively this way. This means that in these species the neoblasts completely take over the function of the germline. Therefore, at least a part of the soma of these *Planaria* has to be non-aging. This “somatic immortality” can be illustrated by experiments in which the injection of a single neoblast was sufficient to save individuals in which all cycling cells were killed by irradiation and who then went on to establish new populations (Wagner et al., 2011). Stem cell aging is a major reason why organs and tissues in other species become less and less able to perform their tasks over time. It is therefore plausible that mechanisms associated with this process can be avoided in *Planaria*. In this context, it has been shown, e.g., that continuous telomere-shortening is avoided in the soma of asexual *Planaria* because telomerase is up-regulated in regeneration (Tan et al., 2012). On the other hand, the main biological function of cellular senescence is tumor suppression in tissues affected by somatic mutations (van Deursen, 2014). Therefore, it is often assumed that *Planaria* must have a high genomic stability and/or efficient DNA repair mechanisms (Sahu et al., 2017; Barghouth et al., 2019). In line with that, it was observed that the Piwi-piRNA pathway, which normally protects the germline from transposable element activity, is somatically expressed both in *Planaria* and *Hydra* (Sturm et al., 2017). More research is needed to find out whether activation of germline pathways can be a strategy to prevent stem cell aging. High-quality genome transcriptome assemblies are available for the frequently used species *Schmidtea mediterranea* (Grohme et al., 2018; Swapna et al., 2018) that contains both sexual and asexual strains. For the same species a single cell transcriptomics-based cell type atlas was published (Plass et al., 2018). Further genome and transcriptome assemblies are available for several additional species, including *Dugesia japonica* (Nishimura et al., 2012; An et al., 2018), *Procotyla fluviatilis* (Sikes and Newmark, 2013) and *Dendrocoelum lacteum* (Liu et al., 2013). Gene knock-down via RNA interference is well established and often used in *Planaria* (Rouhana et al., 2013). Further valuable tools are irradiation protocols for targeted or complete elimination of an individual’s neoblasts in combination with the possibility of transplantation of tissue or even single neoblasts (Wagner et al., 2011; Rojo-Laguna et al., 2019). As with *Hydra*, one of the great challenges for the future will be to explore applications for transferring the knowledge gained in these invertebrates to humans.

## Discussion

Any selection of non-canonical model organisms is necessarily incomplete. Similar to African mole-rats, blind mole rats (*Spalax*), e.g., are strong positive outliers from the lifespan to body mass correlation (Tacutu et al., 2018) and also extremely cancer resistant – the latter possibly mediated by a concerted necrotic cell death mechanism (Gorbunova et al., 2012). Also, elephants exhibit strong cancer resistance, which was associated with a high number of copies of tumor suppressor TP53 (Sulak et al., 2016) and further tumor suppressors (Vazquez and Lynch, 2021). Ant queens reach extreme lifespans of up to 45 years unexpected for their size and, moreover, usually live many times longer than workers (Giraldo and Traniello, 2014). Deep-sea vestimentiferan tubeworms such as *Lamellibrachia luymesi*, *Seepiophila jonesi* and *Escarpia laminata* can reach lifespans of more than 250 years (Durkin et al., 2017). The “immortal jellyfish” *Turritopsis dohrnii* undergoes a reverse development from the medusa to the actually preceding, more juvenile polyp stage in case of injuries or age weakness due to transdifferentiation. It is assumed that this process can be repeated indefinitely and thus their lifespan, in contrast to most other species, is not intrinsically limited (Piraino et al., 1996). Chameleons of the genus *Furcifer* have highly variable life spans. For example, members of the southern population of the species *F. labordi* are considered the shortest-lived known tetrapods, with mean lifespans of 4-5 months, while parts of the northern population live twice as long and other species of the same genus even live more than 10 times as long (Karsten et al., 2008; Eckhardt et al., 2017; Tacutu et al., 2018). One criterion’ for selecting suitable models was the availability of scientific, age-relevant knowledge on a species or taxon. Nevertheless, we readily concede that the distinction is a matter of interpretation and that other species, as those briefly mentioned above, could possibly have been included.

**FIGURE 3:**
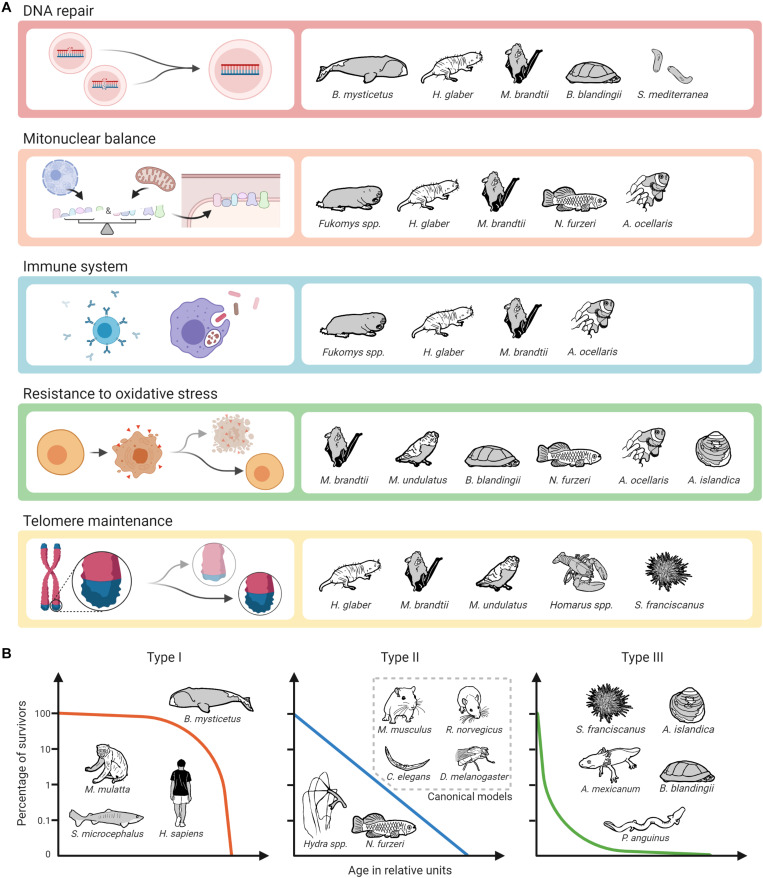
Biological pathways and mechanisms identified as potential contributors to longevity on alternative model organisms of aging and survival curve types. **(A)** The assignment of mechanisms and species does not claim to be complete, as alternative model organisms are not only less well studied than canonical ones, but also not all of the selected mechanisms have so far been addressed in all of the mentioned non-canonical species. Created with BioRender.com. **(B)** The assignment of survival curve types and species can only be done schematically.

Regarding potential molecular targets to slow down the aging process, unsurprisingly, the most promising targets from established (short-lived) model organisms also appear to some extent in the studies conducted in non-canonical species. As described above, this applies, e.g., to the GH/IGF1 axis, the mTOR pathway, and sirtuins (Fontana et al., 2010; Longo et al., 2015; Pan and Finkel, 2017; Duran-Ortiz et al., 2021). It is interesting to note, however, that especially in long-lived alternative model organisms other mechanisms seem to play an even more prominent role. This is particularly true for enhanced DNA repair, for which there is ample evidence from various extremely long-lived mammals, turtles, and *Planaria* that it may be a critical factor in longevity. The same applies to evolutionary adaptations that are associated with the coordination of protein synthesis of nuclear and mitochondrially coded components of the respiratory chain, called mitonuclear balance. Such adaptations have been seen so far in mole-rats, bats, killifishes and clownfishes. Moreover, findings in two long-lived social mole-rat genera (*Heterocephalus* and *Fukomys*) suggest that enhanced proteasome activity contributes to their long life and healthspan. There is also evidence that specific adaptations regarding the immune systems of mole-rats, bats and clownfish significantly contribute to their longevity (Figure 3A). Regarding the impact of oxidative stress and telomere maintenance on aging, however, findings from non-canonical model organisms are similarly ambiguous as those from canonical ones.

Apart from this, our review has not uncovered convergently evolved longevity patterns regarding cellular and molecular mechanisms yet. However, this is not very surprising given that high longevities evolved independently in very different phylogenetic lineages and equally different aquatic, fossorial, terrestrial, or airborne environments. Under so manyfold combinations of phylogenetic and ecological constraints that have shaped the gradual adaptation of the respective genomes, we should not *a priori* expect evolution to have always followed the same paths to reach converging solutions for this complex trait. Therefore, the recurrence of at least some patterns of genetic architecture, as described above, is far from trivial. Most surprising is that adaptations of processes such as mitonuclear balance or resistance to oxidative stress seem to play an important role both for longevity and extremely short lifespans. This suggests that evolutionary trade-offs exist along these processes that allow either adaptation toward selection advantages during the early stages of ontogenesis, e.g., rapid growth in *N. furzeri*, or modification toward longer lifespans. Furthermore, it is perhaps simply too early to expect more such patterns to emerge before these and other non-canonical species have been studied in more detail by a broader aging research community.

Canonical species did not become the workhorses of biological research by accident; their rapid maturity and reproduction make them economical to breed and rear in captivity. However, their rapid maturity and short lives are the result of millions of years of adaptation to a very different evolutionary landscape than that of the long-lived species we have reviewed above. So, although it made sense to first explore the cellular and molecular basis of aging in the already well studied, short-lived canonical species, it is time for us to move on if we are to progress.

Without doubt, non-canonical model organisms offer the possibility to study mechanisms relevant for an extended lifespan that do not occur in canonical ones. These include the species-specific causes of cancer resistance, e.g., in bathyergid mole-rats, blind mole-rats and elephants, the capacity of *Hydra* and *Planaria* stem cells to not exhaust over time, the physiological and molecular mechanisms linking longevity and neoteny in amphibians, and likely a wealth of further ones yet to be explored. Like humans, many of our non-canonical model species are adapted to relative safety from predators, either based on their subterranean environment, their special defenses, or their size. Unlike mice, rats, fruit flies and roundworms, with type II survivorship (sensu Pearl and Miner, 1935), many of our non-canonical species have evolved a type I or type III survivorship curve. Type I species typically include top predators or otherwise safe large individuals. We are long-lived because we are relatively safe from predation for our entire lives. Type III species on the other hand suffer much early mortality, but once they grow to a safe size, they can live very long lives. Type II species however are typically what we think of as “prey species” and suffer from predation at a relatively constant pace throughout their relatively short lives. Given that humans evolved under a type I survivorship curve, we might expect to find that we are better suited to exploit the longevity adaptations discovered in whales (such as DNA repair), but less with type III species such as giant clams (resistance to oxidative stress). On the other hand, perhaps we may be able to exploit a wide array of strategies to promote longevity as, e.g., bats apparently can (Figure 3B). One thing is certain, we will not discover the details of those strategies by restricting our work to the short-lived canonical species.

## Conclusion

We summarized the specific characteristics of a broad taxonomic range of alternative animal models suitable for elucidating processes involved in delayed and accelerated senescence in terms of life expectancy, pre-existing discoveries, and available data and resources. The species presented have exceptional lifespans, an enormous regeneration potential or a remarkable resistance to aging-related diseases. Previous findings on the possible molecular causes confirm the mechanisms known from classical model organisms, but with a different weighting of the various known aging-related signaling pathways. In addition, there are some intriguing, life-prolonging mechanisms to which no equivalent in classical model organisms exists. Including this wealth of evolutionary adaptations into future research will most likely broaden our understanding of the aging process and may eventually contribute to the development of interventions for granting humans a longer and healthier lifespan.

## Author Contributions

AS und SuH conceived and outlined the manuscript. SuH, AS, EG, SB, AC, PD, TH, AH, StH, PK, and ET wrote and edited the original draft. Graphs were prepared by SH and PK. SB and SuH did the final editing. All authors contributed to the article and approved the submitted version.

## Conflict of Interest

The authors declare that the research was conducted in the absence of any commercial or financial relationships that could be construed as a potential conflict of interest.

## Footnotes

1 https://www.proteusgenome.com/
